# STVNa Attenuates Isoproterenol-Induced Cardiac Hypertrophy Response through the HDAC4 and Prdx2/ROS/Trx1 Pathways

**DOI:** 10.3390/ijms21020682

**Published:** 2020-01-20

**Authors:** Fei Liu, Hao Su, Bo Liu, Ying Mei, Qingjin Ke, Xiaoou Sun, Wen Tan

**Affiliations:** Institute of Biomedical and Pharmaceutical Sciences, Guangdong University of Technology, Guangzhou 510006, China; 1111706007@mail2.gdut.edu.cn (F.L.); hsu@cuhkri.org.cn (H.S.); 2111806124@mail2.gdut.edu.cn (B.L.); 1111706012@mail2.gdut.edu.cn (Y.M.); kejingwa@gdut.edu.cn (Q.K.)

**Keywords:** cardiac hypertrophy, ROS, thioredoxin, peroxiredoxin, isosteviol sodium

## Abstract

Recent data show that cardiac hypertrophy contributes substantially to the overall heart failure burden. Mitochondrial dysfunction is a common feature of cardiac hypertrophy. Recent studies have reported that isosteviol inhibits myocardial ischemia-reperfusion injury in guinea pigs and H9c2 cells. This work investigated the protective mechanisms of isosteviol sodium (STVNa) against isoproterenol (Iso)-induced cardiac hypertrophy. We found that STVNa significantly inhibited H9c2 cell and rat primary cardiomyocyte cell surface, restored mitochondrial membrane potential (MMP) and morphological integrity, and decreased the expression of mitochondrial function-related proteins Fis1 and Drp1. Furthermore, STVNa decreased reactive oxygen species (ROS) levels and upregulated the expression of antioxidant factors, Thioredoxin 1 (Trx1) and Peroxiredoxin 2 (Prdx2). Moreover, STVNa restored the activity of histone deacetylase 4 (HDAC4) in the nucleus. Together, our data show that STVNa confers protection against Iso-induced myocardial hypertrophy primarily through the Prdx2/ROS/Trx1 signaling pathway. Thus, STVNA is a potentially effective treatment for cardiac hypertrophy in humans.

## 1. Introduction

Cardiac hypertrophy has traditionally been associated with poor prognosis. It is also among the key diseases that cause heart failure and other conditions, such as hypertension and ischemic heart disease. Two-dimensional (2D) longitudinal and radial strain have been identified as an echocardiographic marker to study hypertrophic cardiomyopathy (HCM) patients and their relatives. Radial strain and particularly basal inferior and posterior segmental reduction used to identify mutation carriers in the pre-clinical phase of disease [1]. The development of heart failure includes various processes that lead to the expression of fetal genes such as β-myosin heavy chain (β-MHC) and atrial natriuretic peptide (ANP), which ultimately enlarges the cardiomyocyte surface area [2]. Cardiomyocytes contain numerous mitochondria for sufficient Adenosine Triphosphate (ATP) generation to drive energy-dependent cardiac processes. Mitochondrial fission is among the key events that regulate cell function and is related to cardiac hypertrophy [3,4,5]. Mitochondrial fission 1 (Fis1) and cytosolic protein dynamin-related protein 1 (Drp1) participate in cell apoptosis and mitochondrial fragmentation [6,7]. Therefore, further research into the mechanisms of mitochondrial fission in cardiac hypertrophy may lead to new ideas for developing effective curative approaches for cardiac hypertrophy and other related diseases.

Many clinical interventions are available to attenuate or inhibit cardiac hypertrophy. Excessive reactive oxygen species (ROS) plays a vital role in myocardial hypertrophy [8,9]. ROS will activate various hypertrophic signaling kinases which eventually cause cell apoptosis and cardiac remodeling [10,11,12,13]. Therefore, inhibiting or eliminating excessive ROS production may prevent the development of cardiac hypertrophy [14]. The status of reduction/oxidation (redox) processes has a profound impact on cardiac syndromes. Thioredoxin 1 (Trx1) and Peroxiredoxin 2 (Prdx2) are ROS-scavenging enzymes that regulate redox signaling. These antioxidant proteins can eliminate excessive cellular ROS or reduce substrate proteins oxidized by ROS.

Trx1 belongs to the thioredoxin protein group, which localizes to both the cytosol and nucleus of heart cells [15,16,17,18]. Overexpression of Trx-1 in mice reduced cardiac hypertrophy induced by aortic banding, while Trx-1 deficiency resulted in cardiac hypertrophy and increased oxidative stress under baseline conditions [19]. Prdx2 is a ubiquitous cytosolic peroxidase, a member of the typical 2-Cys peroxiredoxin enzyme family that protects cells by eliminating cellular H_2_O_2_ [20,21,22]. As a substrate of Prdx2, Trx1 maintains a steady state of cell redox [23]. Previous studies have demonstrated that Trx1 and Prdx2 expression were downregulated under hypertrophic conditions [23,24]. In addition, HDAC4, a class II histone deacetylase, modulates cardiac hypertrophy. It was reported that Trx1 inhibited the translocation of HDAC4 and then blocked cardiac hypertrophy signals [25]. Considering the important role of Prdx2 and Trx1 in hypertrophied heart, therapies aimed at inhibiting Prdx2 and Trx1 are likely to be effective for the prevention of cardiac hypertrophy.

Isosteviolis is a bioactive extract of stevioside with anti-hypertension, antioxidant, anti-inflammatory, anti-hyperglycemia and anti-tumor properties [26]. Isosteviol was shown to exert a cardioprotective effect in ischemia-reperfusion injury in various animal models [27]. Isosteviol sodium (STVNa) is a soluble sodium salt of isosteviol, previously demonstrated by our group to confer neuroprotection after acute focal cerebral ischemia in rats [28]. However, the effect of isosteviol or STVNa on cardiac hypertrophy remains poorly understood. Therefore, we established a model of cardiac hypertrophy using Iso stimulation in vitro and in vivo to study the cardioprotective effect of STVNa and its underlying mechanism. Specifically, we asked if STVNa can restore mitochondrial function via regulation of HDAC4 and the Prdx2/ROS/Trx1 axis.

## 2. Results

### 2.1. STVNa Reversed Cardiomyocyte Hypertrophy Induced by Iso

**Figure 1:**
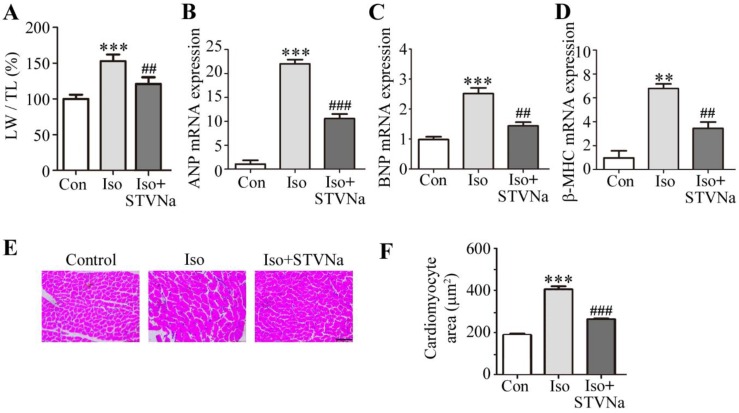
Cellular effects of STVNa after Iso-induced cardiac hypertrophy in vivo. Rats were allocated into three groups (*n* = 8): the control group received 0.9% NaCl injections; the Iso group received subcutaneous injections of 5 mg/kg/d Iso for 7 consecutive days to create a model of cardiac hypertrophy; and the Iso + STVNa group received Iso (5 mg/kg) and isosteviol sodium (4 mg/kg). (**A**) The LW/TL ratio was calculated. (**B**–**D**) Real-time quantitative PCR results showing mRNA levels of ANP, β-MHC and BNP. (**E**) Hematoxylin -eosin (HE)-staining results of left ventricle cross-sections. Scale bar: 50 μm. (**F**) Determination of cardiomyocyte surface area by HE staining. The values shown in this graph represent means ± SD. of data from three independent experiments (*n* = 30). ** *p* < 0.01, *** *p* < 0.001 versus the control group; ^##^ *p* < 0.01, ^###^ *p* < 0.001 versus the Iso group. Iso, isoproterenol; BNP, brain natriuretic polypeptide; β-MHC, β-myosin heavy chain; ANP, atrial natriuretic peptide.

Iso is a non-specific β-adrenergic receptor agonist commonly used to induce cardiac hypertrophy [29]. Sprague-Dawley rats (*n* = 8) were injected with Iso at 5 mg/kg/d for 7 days to establish an in vivo model of cardiac hypertrophy. As shown in Figure 1, STVNa attenuated the Iso-induced changes. The LW/TL ratio was larger in Iso-treated rats than in the control group, and the HE staining revealed typical hypertrophic changes. The cardiomyocyte cross-sectional area was significantly increased, and co-treatment with STVNa decreased the LW/TL ratio (Figure 1A). The cardiomyocyte cross-sectional area (*n* = 30) tended to decrease in the left ventricle (Figure 1E,F). Iso-treated rats showed high expression of atrial natriuretic peptide (ANP), β-myosin heavy chain(β-MHC) and brain natriuretic polypeptide (BNP), whereas the level of these biomarkers was decreased in rats treated with STVNa (Figure 1B–D).

**Figure 2:**
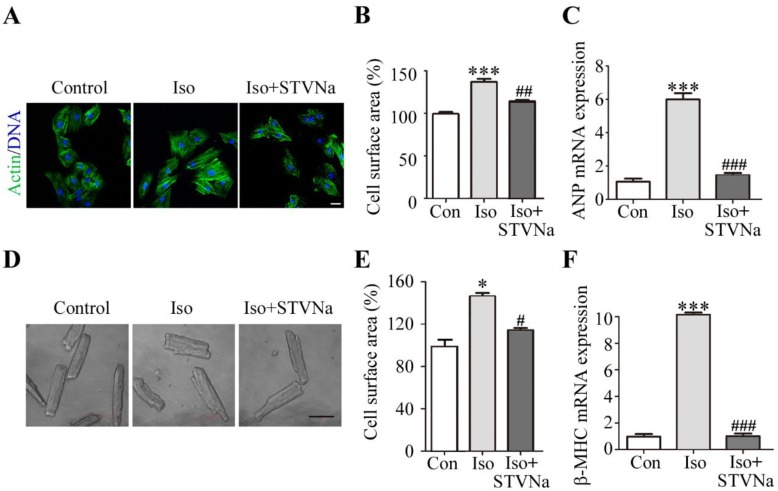
STVNa inhibited cardiomyocyte hypertrophy induced by Iso in vitro. H9c2 cells were cultured in Dulbecco’s Modified Eagle Medium (DMEM) which containing 1% fetal bovine serum (FBS) under the following conditions: control, DMEM only; Iso, 10 μmol/L Iso; Iso + STVNa, 10 μmol/L Iso and 5 μmol/L STVNa. (**A**) The surface area of H9c2 cells determined by rhodamine-phalloidin staining in all groups (**B**, *n* = 30). Scale bar: 50 μm. (**C**) ANP mRNA expression in H9c2 cells. (**D**) The cardiomyocytes were isolated from the rat heart (*n* = 5) and the surface area was visualized by a microscope (scale bar: 50 μm). The representative cell surface area in each group was calculated (**E**, *n* = 30). (**F**) The mRNA level of β-MHC in H9c2 cells. The values shown in this graph represent means ± S.D. from three independent experiments. * *p* < 0.05, *** *p* < 0.001 versus the control group; ^#^ *p* < 0.05, ^##^ *p* < 0.01, ^###^ *p* < 0.001 versus the Iso treatment group.

To explore the effect of STVNa on Iso-induced cardiac hypertrophy in vitro, we established a research model using the H9c2 cell line by exposing these cells to 10 µm Iso for 48 h. This led to a significant increase in cell surface area of H9c2 cells (*n* = 30), which was reversed by STVNa treatment (Figure 2A,B). Consistent with the results obtained from the H9c2 cells, we found that STVNa significantly reduced the cell surface area of primary rat cardiomyocytes (*n* = 30) isolated from the rat heart after Iso injection (Figure 2D,E). Our results also showed that STVNa significantly reduced the increase in ANP and β-MHC mRNA expression induced by Iso (Figure 2C,F). The above results suggested that STVNa may inhibit Iso-induced hypertrophy.

### 2.2. Effect of STVNa on Mitochondrial Integrity After Iso Induction

**Figure 3:**
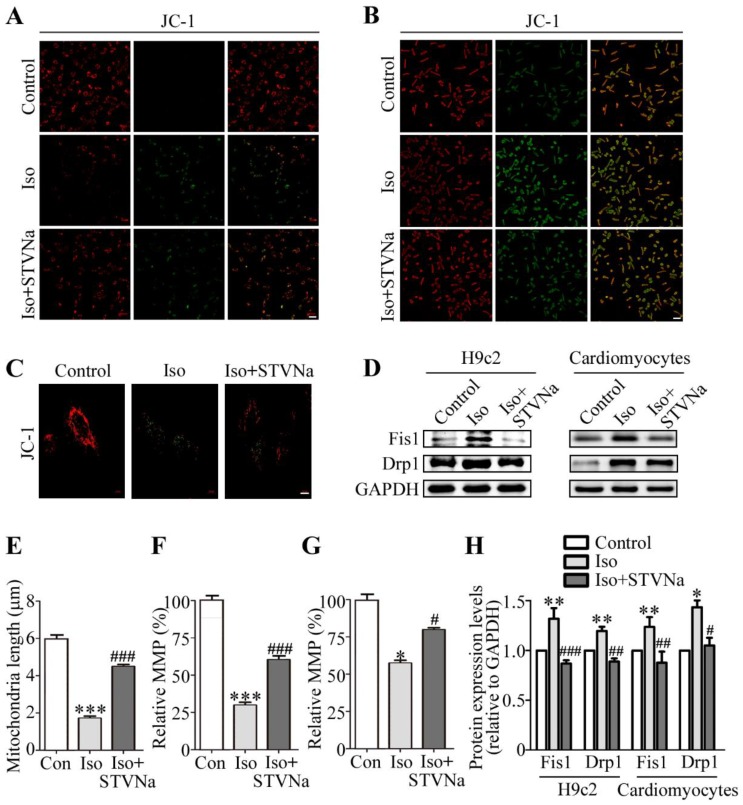
Effect of STVNa on mitochondrial integrity after Iso induction. Cells were seeded in 24-well plates and treated with JC-1 at 37 °C for 20 min to measure the Mitochondrial Membrane Potential (MMP). Confocal micrographs of JC-1 staining in H9c2 cells (**A**) and primary cardiomyocytes (**B**). Scale bar: 50 μm. (**C**) Mitochondrial morphology (scale bar: 10 μm) showing the length of mitochondria (**E**, *n* = 15). The results for relative mitochondrial membrane potential (MMP) were calculated using Zeiss 2012 imaging processing and 30 cells in each group were included. Data are shown as percentages of the control level. Images are representative of three independent experiments. 30 μg of total protein was used and the relative protein expression level were expressed as the ratio of target protein/ glyceraldehyde-3-phosphate dehydrogenase (GAPDH). (**D**) Western blot analysis and quantitative results (**H**) of mitochondrial fission-related proteins Fis1 and Drp1 in cardiomyoctyes and H9c2 cells. (**F**,**G**). The values shown in this graph represent means ± S.D. of data from three independent experiments. * *p* < 0.05, ** *p* < 0.01, *** *p* < 0.001 versus the control group; ^#^ *p* < 0.05, ^##^ *p* < 0.01, ^###^ *p* < 0.001 versus the Iso treatment group.

A key feature of a failing mitochondrion is the diminished mitochondrial membrane potential (MMP). In this study, 10 μM JC-1 solution was used to measure the MMP at 37 °C for 20 min. As shown in Figure 3A (in H9c2 cells) and 3B (in isolated primary rat cardiomyocyte), the MMP was significantly downregulated in the Iso-induced group. After treatment with STVNa, the ratio partially recovered and the effect of Iso was significantly inhibited (Figure 3F,G). The results for relative mitochondrial membrane potential (MMP) were calculated by Zeiss 2012 imaging processing and 30 cells in each group were included. We also examined structural alterations to the mitochondria. In the control group, mitochondria of H9c2 cells appeared tubular as evidenced by JC-1 staining (Figure 3C), but after Iso treatment, the mitochondria became small and fragmented, while treatment with STVNa restored mitochondrial morphology to some extent. We then calculated the length of the mitochondria in different groups and assessed the protein level of Fis1 and Drp1 as indicators of mitochondrial fission. Figure 3D (in H9c2 cells) and 3H (in primary rat cardiomyocytes) reveal that compared to the control cells, Fis1 and Drp1 were highly expressed after Iso treatment (*p* < 0.05). However, when co-cultured with STVNa, the level of Drp1 and Fis1 was decreased (*p* < 0.05) and the length of the mitochondria was restored significantly (Figure 3E, *n* = 15). Thus, we suggest that STVNa inhibits Iso-induced cardiomyocyte hypertrophy by regulating the expression of mitochondrial fission and fusion proteins.

### 2.3. STVNa Inhibited ROS Burst and Increased Anti-Oxidant Trx1 and Prdx2 Expression

**Figure 4:**
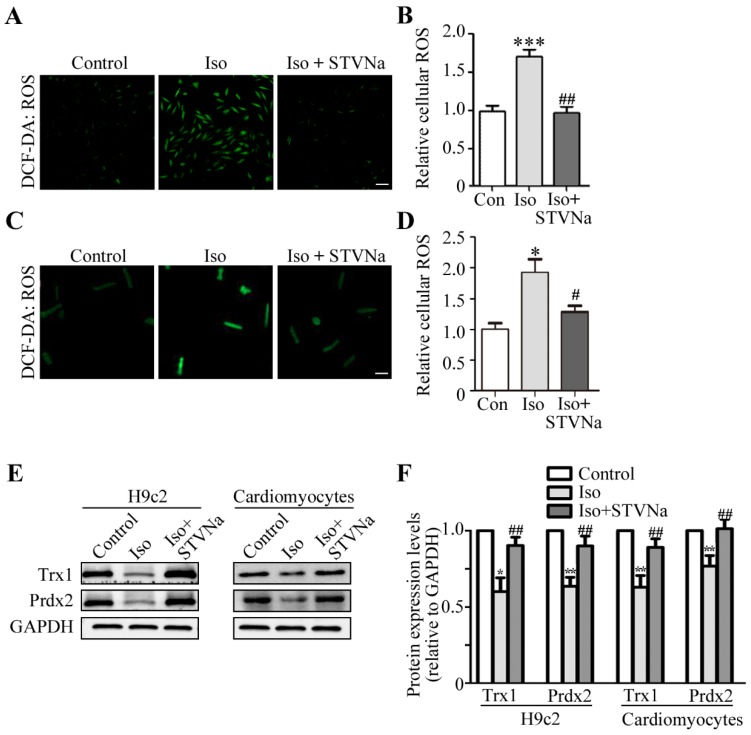
STVNa inhibited Iso-induced ROS generation, Trx1 and Prdx2 expression in H9c2 cells and rat cardiomyocytes. In total, 10 μM DCFH-DA was used to measure the intracellular ROS. Confocal images showing ROS levels in H9c2 cells (**A**) and cardiomyocytes (**C**). The micrographs are representative of three independent experiments. Scale bar: 50 μm. (**B**,**D**) Graphs of ROS levels in H9c2 cells (**B**, *n* = 30) and cardiomyocytes (**D**, *n* = 30). In total, 30 μg of total protein was extracted and the western blot was performed. (**E**) Immunoblotting showing the expression of Prdx2 and Trx1 in H9c2 cells and cardiomyocytes and the relative fold change in protein levels of Trx1 and Prdx2 compared to β-actin (**F**). The values shown in this graph represent means ± S.D. of data from three independent experiments. * *p* < 0.05, ** *p* < 0.01, *** *p* < 0.001 versus the control group; ^#^ *p* < 0.05, ^##^ *p* < 0.05 versus the Iso treatment group.

Several cardiac diseases, such as heart failure, hypertrophy, I/R injury and atherosclerosis, are associated with mitochondrial dysfunction. ROS is a key modulator of mitochondrial disorders [4]. This led us to test whether STVNa could prevent Iso-induced cardiac hypertrophy using the 5-(and-6)-Carboxy-2′,7′-Dichlorofluorescein Diacetate (DCFH-DA) assay to measure intracellular ROS production in H9c2 cells (Figure 4A) and rat cardiomyocytes (Figure 4C). The DCFH-DA assay was performed and the confocal micrographs were taken. Figure 4A–D reveals that the ROS level increased significantly after Iso treatment of H9c2 cells and rat cardiomyocytes, while STVNa co-treatment markedly decreased the quantity of ROS fluorescence relative to the Iso-treated groups (*n* = 30).

**Figure 5:**
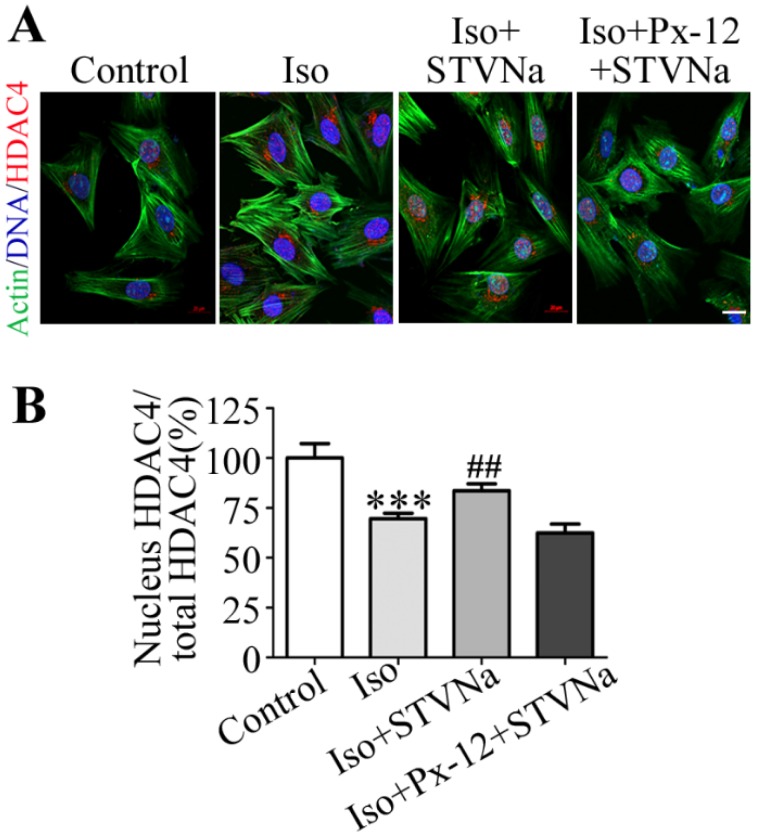
STVNa inhibited the translocation of HDAC4 from the nucleus to the cytoplasm in H9C2 cells. (**A**) IF staining showing HDAC4 shuttling between the cytoplasm and nucleus. Green: rhodamine-phalloidin; red: HDAC4; blue: 4′, 6-diamidino-2-phenylindole (DAPI). Representative images from four independent experiments. Scale bar: 20 μm. (**B**) The ratio of nuclear HDAC4 to total HDAC4. The results were calculated using Zeiss 2012 imaging processing and 30 cells in each group were included. The values shown in this graph represent means ± S.D. of data from three independent experiments. *** *p* < 0.001 versus the control group; ^##^ *p* < 0.01 versus the Iso treatment group.

Prdx2 and Trx1 have been reported to be antioxidant factors in mammalian tissues. To understand how STVNa inhibited the ROS burst, we investigated the effects of STVNa on protein levels of Trx1 and Prdx2. In total, 30 μg of total protein was used and the relative protein expression level were expressed as the ratio of target protein/GAPDH. As shown in Figure 5E,F, the expression of Trx1 and Prdx2 markedly decreased in the Iso-treated group, but co-treatment with STVNa reversed this effect in H9c2 cells and cardiomyocytes when compared to the Iso group. Thus, we reasoned that STVNa treatment prevented cardiac hypertrophy by downregulating Prdx2 and Trx1 expression.

### 2.4. STVNa Inhibits Iso-Induced Cardiac Hypertrophy by Regulating Nuclear Translocation of HDAC4

The translocation of class II histone deacetylases (HDACs) from the nucleus to the cytoplasm has been reported to be the terminal stage leading to cardiac hypertrophy [30]. Trx1 prevents cardiac hypertrophy by restoring the nuclear localization of class II HDAC4 oxidized [17]. The exact roles of Prdx2 and Trx1 during cardiac hypertrophy are not completely known. In this study, Immunofluorescence (IF) staining was used and our data showed that the ratio of nuclear HDAC4 to total HDAC4 was decreased in the Iso-induced group, and STVNa co-treatment increased this ratio. Furthermore, the ratio was not significantly different between Iso group and cells treated with PX-12 (a Trx1 inhibitor). The results were calculated by Zeiss 2012 imaging processing (*n* = 30). These findings suggest that STVNa inhibits Iso-induced cardiac hypertrophy by regulating the translocation of HDAC4 from the nucleus to the cytoplasm; inhibition of Trx1 prevents this effect.

## 3. Discussion

The development of cardiac hypertrophy is considered to be an adaptive response to preserve the heart’s function following persistent stimulation. It may, however, lead to detrimental effects such as heart failure if it becomes de-compensated. Our previous studies provided evidence that STVNa confers protection against ischemia-reperfusion injury in H9c2 cells [31] and isolated ventricular myocytes [32]. Therefore, this study tested whether the protection by STVNa extends to Iso-induced cardiac hypertrophy.

Our results showed that Iso treatment significantly upregulated mRNA levels of the hypertrophic genes ANP, β-MHC and BNP, and also increased cardiomyocyte cell surface area, confirming that chronic exposure to Iso induced hypertrophy in H9c2 cells and rat cardiomyocytes. However, co-culture with STVNa abrogated the upregulating effects of Iso on ANP, β-MHC and BNP expression, indicating that STVNa suppresses pro-hypertrophic signals caused by Iso.

The mitochondrion is a vital organelle in cardiomyocytes [3]. MMP reflects the status of mitochondrial function, the measurement of which is essential for understanding the molecular mechanisms controlling cardiomyocyte function [33]. Mitochondrial dysfunction, therefore, modifies the occurrence of cardiac hypertrophy [34]. In the present study, STVNa inhibited the protein levels of Fis1 and Drp1, and restored mitochondrial morphology and function. In H9c2 cells treated with Iso, levels of MMP decreased [31]. Notably, co-treatment with STVNa abolished the effects of Iso on MMP. Mitochondrial membrane depolarization compromises the respiration function, triggering apoptosis. Normalization of MMP in cells treated with EUK-134 attenuated cardiac hypertrophy [35]. Therefore, we reasoned that STVNa may prevent cardiac hypertrophy by preserving mitochondrial integrity and function.

Similar to other studies [10,36], we observed that cellular ROS signals were upregulated following chronic exposure to Iso. Activation of the Iso hypertrophic signaling pathway leads to the generation of ROS, which acts as a positive feedback signal, further stimulating the hypertrophy signaling pathway and apoptotic signaling. Persistent activation of these pathways eventually causes cell injury [34]. Therefore, we hypothesized that inhibition of cellular ROS generation or decreasing ROS levels may protect cardiomyocytes by improving mitochondrial morphology and function in Iso-induced myocardial hypertrophy. Mitochondria are an important source of ROS, and excessive ROS production may induce mitochondrial injury and exacerbate ROS generation. It has been shown that induction of cardiac hypertrophy by AngII, Tumor necrosis factor -α and α-adrenergic stimuli is ROS-dependent [34]. In the present study, we observed that co-culture with STVNa elevated MMP and enhanced mitochondrial function following Iso-treatment. Exposure of cells to Iso for 48 h increased ROS levels by and STVNa co-culture significantly decreased ROS production, indicating that the protection provided by STVNa was mediated by inhibition of ROS production.

We then explored the effect of STVNa on the expression of anti-oxidant proteins, Trx1 and Prdx2. Trx1 is a multifunctional protein first discovered in both the cytosol and nucleus [17]. Trx1 interacts with ASK-1 to inhibit ASK-1-induced apoptosis, and it was also shown that ROS can activate ASK-1 leading to cardiac hypertrophy [37]. Prdx2 functions as a scavenger of ROS, and can decrease monocyte attachment to endothelial cells and provide cardiac protection against oxidative stress-induced cardiomyocyte apoptosis and death [38]. Histone deacetylation via HDACs is a key process that modifies the reorganization of DNA into chromatin. The transcription of hypertrophy-related genes, such as pro-hypertrophic ANP and anti-hypertrophic GSK3β, requires relaxation of chromatin. Class I and II HDACs regulate the development of cardiac hypertrophy. Class I HDACs are pro-hypertrophic whereas Class II HDACs prevent hypertrophy. HDAC4 is a member of the class II HDACs which shuttles between nucleus and cytoplasm to modulate cardiac hypertrophy [15]. It was reported that Trx1 can inhibit the translocation of HDAC4 and further repress the cardiac hypertrophy signals, MEF2 and NFAT [1]. In the current study, we found that Trx1 protein was downregulated in cardiac hypertrophy, and this effect was inhibited by STVNa. It has also been found that overexpression of Trx1 in mice inhibited oxidative stress-induced cardiac hypertrophy and that inhibition of endogenous thioredoxin aggravated oxidative stress and cardiac hypertrophy [1,39]. Thus, we suspect that the cardiac protection provided by STVNa against Iso-induced cardiac hypertrophy may be correlated with the upregulation of Trx1. Additionally, Trx1 can also clear the overload of ROS in cells and inhibit cell injury caused by ROS [19]. Based on our results, we reasoned that STVNa may upregulate Trx1 expression to inhibit ROS overload and ROS-induced cell injury. Indeed, co-culture with STVNa increased Trx1 expression and HDAC4 translocation to the cytoplasm, thereby preventing Iso-induced cardiac hypertrophy. We had previously observed that STVNa increased the sensitivity of sarcKATP channels, the activation of which has been identified as a critical step in cardiac protection [40]. However, we still do not understand how STVNa increases the sensitivity of sarcKATP. We speculate that STVNa does so by elevating the expression of Trx1 and preserving MMP, since Trx1 can increase KATP opening sensitivity and inhibit the downregulation of MMP [19]. It is also known that Prdx2 downregulates Iso-induced cardiac hypertrophy [21]. In our study, we observed that STVNa elevated Prdx2 protein expression, while Iso treatment inhibited the expression of Prdx2. We reason that the increase in ROS generation may be a result of the decreased scavenging effect of Prdx2 following Iso exposure. Therefore, STVNa may also exert protection by elevating Prdx2 expression.

## 4. Materials and Methods

### 4.1. Materials

The phalloidin, JC-1 and DCFH-DA probes were bought from Life Technology (St. Louis, MO, USA). PX-12, DAPI and isoprenaline hydrochloride (Iso) were purchased from Sigma-Aldrich (USA). Rhodamine-conjugated secondary antibodies were obtained from Jackson ImmunoResearch Laboratories (West Grove, PA, USA). STVNa was obtained from Chemical Development Laboratories of Key Biological Pharmaceutical Company (Dongguan, China). Trizol was obtained from Generay (Shanghai, China). Antibodies against Drp1, Trx1, Prdx2 and GAPDH were acquired from Cell Signaling Technology (Beverly, MA, USA). HDAC4 was obtained from Abcam (Cambridge, MA, UK). The Fis1 antibody and HRP-conjugated secondary antibodies were acquired from Santa Cruz Biotechnology (Santa Cruz, CA, USA).

### 4.2. Animal Experiments and Primary Cardiomyocyte Isolation

In vivo experiments were performed using male Sprague-Dawley rats weighing 200–220 g (Certification No. 44008500011386, SPF grade) housed at Sun Yat-Sen University (Guangzhou, China). This study was approved by the Institutional Animal Care and Research Advisory Committee of the Institute of Biomedical & Pharmaceutical Sciences at Sun Yat-Sen University (permit number 20140515171141; approved 15 May 2014). The rats were reared in cages and allowed free access to water under a humidified atmosphere (55 ± 5%) controlled to a temperature of 22 ± 2 °C and were fed standard chow 1 week prior to experimentation. Rats were allocated to three groups: the control group received 0.9% NaCl injections; the Iso group received subcutaneous injections of 5 mg/kg/d Iso for 7 consecutive days to create a model of cardiac hypertrophy; and the Iso + STVNa group received Iso (5 mg/kg) and isosteviol sodium (4 mg/kg). After 7 days, the animals were euthanatized by cervical dislocation and the left ventricular weight (LW) was measured. Hearts were isolated and washed. The blood and other liquids were cleaned from the heart using filters. The tibia length (TL) was measured using electronic scales and the ratio of LW to TL (LW/TL) was calculated. Three rat hearts in each group were prepared for hematoxylin/eosin (HE) staining as follows: after isolation, they were fixed in 10% formalin, embedded in paraffin and cut transversely into 3 μm-thick sections. The other rat hearts were digested to isolate primary rat cardiomyocytes. The isolation method was described previously [41].

### 4.3. H9c2 Culture and Interventions

H9c2 cells bought from the Chinese Academy of Sciences (Shanghai, China) were cultured in DMEM which contained 15% FBS (Gibco, GrandIsland, NY, USA) under 5% CO_2_ at 37 °C. After 24-h starvation, cells were cultured in 1% FBS/DMEM under the following conditions: control, DMEM only; Iso, 10 μmol/L Iso, Iso + STVNa, 10 μmol/L Iso and 5 μmol/L STVNa, as described previously [31,42].

### 4.4. Measurement of Cell Surface Area

For measurement of cell surface area, cells were seeded in 24-well plates at a density of 5 × 10^3^ cells per well. They were then fixed in 4% paraformaldehyde and permeabilized using 0.1% Triton X-100. After staining with DAPI for 10 min and phalloidin for 40 min, they were imaged with a Zeiss LSM800 confocal microscope and analyzed using Image J. Three repeated measurements were obtained for each group.

### 4.5. ROS Measurement

The DCFH-DA assay was performed to quantify the generation of cellular ROS. Briefly, 1 × 10^4^ cells were grown at 37 °C in 24-well plates and then treated with 10 μM DCFH-DA dissolved in DMEM or Tyrode’s solution for 40 min. Next, they were washed three times with phosphate buffer saline (PBS). Finally, ROS intensity was detected using the Zeiss LSM800 fluorescence microscope at 520 nm emission and 488 nm excitation. This test was performed three times for each group and treatment.

### 4.6. Measurement of Mitochondrial Membrane Potential

H9c2 cells were cultured in 35-mm confocal plates at a density of 2 × 10^4^ cells/well. Thereafter, they were treated with 10 μM JC-1 solution at 37 °C for 20 min. This was followed by washing three times with PBS and suspension in DMEM or Tyrode’s solution for examination. The green monomers and red J-aggregates were observed by a Zeiss LSM800 fluorescence microscope. The mitochondrial membrane potential was expressed as the ratio of red to green fluorescence. This test was performed three times for each group and treatment.

### 4.7. Real-Time quantitative PCR

Total RNA was isolated by treating cells with Trizol reagent (Generay, Shanghai, China) following the manufacturer’s procedures. cDNA was synthesized from 1 µg of total RNA for real-time PCR using ChamQTM SYBR^®^ qPCR Master Mix (Vazyme, China) on the 7500 Real-Time PCR System (Applied Biosystems, Forster City, CA, USA). The mRNA levels of GAPDH, ANP, β-MHC and BNP were measured using GAPDH as the reference gene. The specific primers used to amplify these genes were previously described [31]. This test was performed three times for each group and treatment.

### 4.8. Western Blot Analysis

Western blot was performed as detailed in a previous study [43]. Briefly, total protein was extracted and the protein concentration was measured using a BCA kit (Pierce, Rockford, IL, USA). Next, 30 g protein was resolved by 12% SDS-PAGE and then transferred to 0.22-μm PVDF membranes (Millipore, Billerica, MA, USA). The membranes were blocked and incubated with primary antibodies and then with HRP-conjugated secondary antibodies. The primary antibodies against GAPDH, Fis1, Drp1, Prdx2 and Trx1 were used at 1:2000 dilution. The proteins were visualized with enhanced chemiluminescence detection reagent (Tanon, China). Relative protein expression levels were expressed as the ratio of target protein/GAPDH. This test was performed three times for each group and treatment.

### 4.9. Immunofluorescence (IF) Assay

After treatment, H9c2 cells were seeded in 35-mm confocal plates and fixed. Next, the cells were permeabilized and blocked with 2.5% BSA for 1 h at room temperature. This was followed by incubation overnight with a primary antibody against HDAC4 (diluted 1:50) at 4 °C. Thereafter, the cells were incubated with rhodamine-conjugated secondary antibody (diluted 1:200) at room temperature for 2 h. After staining with phalloidin for 40 min and DAPI for 10 min, immunofluorescence was quantified with a confocal microscope (Zeiss LSM800).

### 4.10. Statistical Analysis

Group comparisons were performed using Student’s t-test, and all data are presented as means ± standard deviations (S.D.). *p* values < 0.05 were considered significant.

## 5. Conclusions

**Figure 6:**
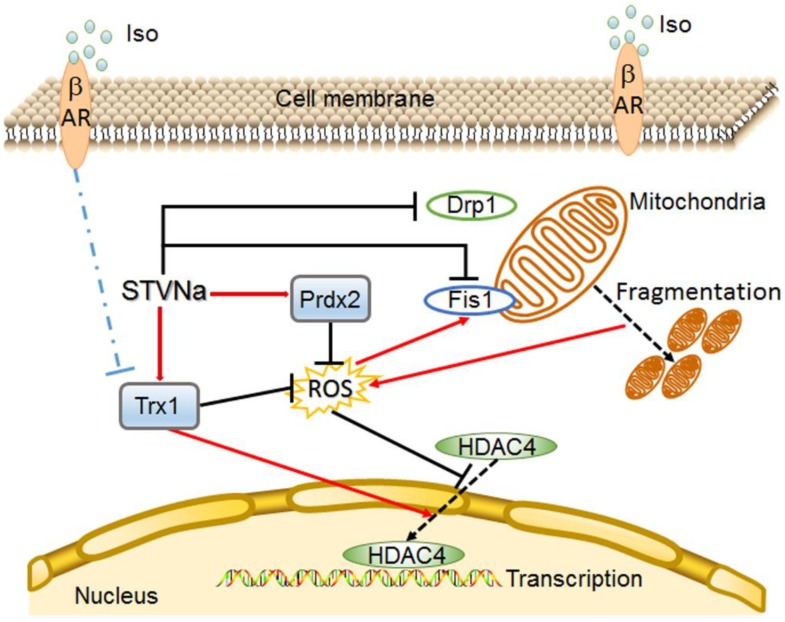
Mechanistic illustration of the effects of STVNa on Iso-induced cardiac hypertrophy. STVNa elevates Trx1 and Prdx2 expression, inhibits cellular ROS overload and ROS-induced cell mitochondrial fission, thus restoring MMP and mitochondrial morphology. This in turn decreases ROS expression. In addition, Trx1 reciprocally inhibits the translocation of HDAC4 from the nucleus to the cytoplasm, thus inhibiting cardiac hypertrophy induced by Iso. STVNa: isosteviol sodium; βAR: β-adrenergic receptor; Iso: Isoprenaline; Prdx2: Peroxiredoxin 2; Trx1: Thioredoxin 1; Fis1: mitochondrial fission 1; Drp1: Dynamin-related protein 1; HDAC4: histone deacetylase 4.

In conclusion, our results demonstrated that STV-Na exerted significant cardioprotective effects against Iso-induced cardiac hypertrophy in H9c2 cells and in primary rat cardiomyocytes, and the possible mechanism may be associated with anti-oxidation and inhibition of HDAC4 translocation from the nucleus to the cytoplasm (Figure 6). These findings provide a new insight into the effect of STV-Na on heart diseases, but further research will be required to explore the specific molecular/causal pathways underlying the effect of STV-Na on cardiac hypertrophy.
